# Learning vector quantization as an interpretable classifier for the detection of SARS-CoV-2 types based on their RNA sequences

**DOI:** 10.1007/s00521-021-06018-2

**Published:** 2021-04-27

**Authors:** Marika Kaden, Katrin Sophie Bohnsack, Mirko Weber, Mateusz Kudła, Kaja Gutowska, Jacek Blazewicz, Thomas Villmann

**Affiliations:** 1 University of Applied Sciences Mittweida, Technikumplatz 17, 09648 Mittweida, Germany; 2 Saxon Institute for Computational Intelligence and Machine Learning, Technikumplatz 17, 09648 Mittweida, Germany; 3 Institute of Computing Science, Poznan University of Technology, Piotrowo 2, 60-965 Poznan, Poland; 4 Institute of Bioorganic Chemistry, Polish Academy of Sciences, Noskowskiego 12/14, 61-704 Poznan, Poland; 5 European Centre for Bioinformatics and Genomics, Piotrowo 2, 60-965 Poznan, Poland

**Keywords:** Learning vector quantization, Interpretable models, Genomic sequence analysis, Reject options

## Abstract

We present an approach to discriminate SARS-CoV-2 virus types based on their RNA sequence descriptions avoiding a sequence alignment. For that purpose, sequences are preprocessed by feature extraction and the resulting feature vectors are analyzed by prototype-based classification to remain interpretable. In particular, we propose to use variants of learning vector quantization (LVQ) based on dissimilarity measures for RNA sequence data. The respective matrix LVQ provides additional knowledge about the classification decisions like discriminant feature correlations and, additionally, can be equipped with easy to realize reject options for uncertain data. Those options provide self-controlled evidence, i.e., the model refuses to make a classification decision if the model evidence for the presented data is not sufficient. This model is first trained using a GISAID dataset with given virus types detected according to the molecular differences in coronavirus populations by phylogenetic tree clustering. In a second step, we apply the trained model to another but unlabeled SARS-CoV-2 virus dataset. For these data, we can either assign a virus type to the sequences or reject atypical samples. Those rejected sequences allow to speculate about new virus types with respect to nucleotide base mutations in the viral sequences. Moreover, this rejection analysis improves model robustness. Last but not least, the presented approach has lower computational complexity compared to methods based on (multiple) sequence alignment.

**Supplementary Information:**

The online version contains supplementary material available at 10.1007/s00521-021-06018-2.

## Introduction

The coronavirus disease 2019 (COVID-19) caused by SARS-CoV-2 viruses, whose origin lies probably in Wuhan (China), is a severe respiratory disease [1]. Currently (May 2020), it is spreading rapidly all over the world [88]. Yet there are several indicators that its molecular characteristics evolve during time [2, 89]. This evolution is mainly driven by mutations, which play an essential role and may be accompanied by mechanisms of stabilization [70, 71].

Therefore, an analysis of virus sequences is essential to understand the spreading and the behavior of the virus population. One aspect is to distinguish several types of the virus, which may force different symptoms and medical conditions. Thus, sequences have to be compared regarding their genomic structure. This can be done by alignment methods or by alignment-free approaches, both coming with pros and cons. Further, the sequences have to be distinguished or classified with respect to their virus types. For this purpose, interpretable models are favored in comparison with black-box approaches like deep networks, because a medical interpretation of the classification decision process is highly desirable. In fact, this could help to detect new virus variants.

### Biological basics regarding SARS-CoV-2

The analysis of the genomic structure by sequencing is currently topic of ongoing research to better understand the molecular dynamics [53]. Obviously, changing the genomic structure may cause new properties and, hence, could increase the difficulties in finding drugs for treatment. For example, changes may lead to behavioral changes, such as the increased binding of the SARS-CoV-2 surface glycoprotein to human ACE2 receptors [37].

Viruses of the family *Coronaviridae* possess a single-stranded, positive-sense RNA genome ranging from 26 to 32 kilobases in length and frequently are extremely similar [44]. Therefore, the analysis of those sequences to understand the genetic evolution in time and space is very difficult. This problem is magnified by incorrect or inaccurate sequencing [75]. Further, mutations are not equally distributed across the SARS-CoV-2 genome [28]. The molecular differences in corona virus populations were investigated using phylogenetic trees so far resulting in three clusters which are identified as virus types [23]. Yet, SNP-based radial phylogeny-retrieved trees of SARS-CoV-2 genomes result in five major clades [28]. Generally, a disadvantage of those decision-tree-like approaches is the problem of out-of-sample considerations, i.e., new data cannot easily be integrated [55, 86]. The respective tree has to be reconfigured completely, which frequently leads to major changes in the tree structure [56, 72].

Frequent mutations in SARS-CoV-2 genomes are in the genes encoding the S-protein and RNA polymerase, RNA primase, and nucleoprotein. Applying a sequence alignment and similarity comparison using the Jaccard index, a method for monitoring and tracing SARS-CoV-2 mutations was established in [90]. However, a general mathematical evaluation of similarities is crucial because respective similarity measures only partially reflect all biological aspects of similarity between RNA sequences [87].

Alignment-based methods usually rely on variants of the Levenshtein distance [38], which, however, are computationally costly: $O\left( l_1\cdot l_2\right)$ is the time complexity for both the Needleman–Wunsch algorithm [51] and for the Smith–Waterman algorithm [26, 68], where $l_1$ and $l_2$ are the sequence lengths. Hence, if $l_1=l_2=l$, the complexity is simply $O\left( l^2\right)$. Both approaches solve internally a mathematical optimization problem, i.e., both algorithms belong to the algorithmic class of dynamic programming with high computational complexity.

In case of multiple sequence alignments (MSAs), the dissimilarity problem is NP-hard [31]. Currently used MSA implementations such as ClustalW [73], MAFFT [33], or MUSCLE [18] therefore rely on the progressive alignment technique [20], which reduces the computational complexity to polynomial time [47]. In the example of MUSCLE, the time complexity amounts to $O(N^4 + N \cdot l^2)$ with *N* being the number of sequences and *l* is the uniform sequence length. Other alignment-based methods for SARS-CoV-2 data consider (multiple) longest common subsequences with similar complexity [41].

Therefore, alignment-free alternatives are promising to avoid this algorithmic complexity [7, 8, 83, 84, 87, 91]. Commonly used approaches are *Bag-of-Words* (BoW [67]), information theoretic methods based on the *Kolmogorov–Smirnov complexity* [35] and the related *Normalized Compression Distance* [13, 40]. Recently, similarities based on *Natural Vectors* gained attraction [17, 42, 92]. These methods have in common that the sequences are considered in terms of their statistical properties and distributions of the nucleotides. However, local information like precise nucleotide positions as well as specific motifs is lost. An overview of prominent measures and their behavior for sequence analysis can be found in [94, 95]. The time complexity for this data coding is only $O(N\cdot l)$ and, hence, much lower than for alignment methods.

In the present publication, we investigate whether alignment-free dissimilarities are suitable for the identification of SARS-CoV-2 clusters/classes in combination with *interpretable machine learning methods* for clustering and classification [4, 5]. This we do for two datasets: GISAID data and NCBI data, see Sect. 2.1. For the first one, virus classes (types) were identified by phylogenetic tree analysis in [23], whereas the second one is without class information.

### Motivation to use an interpretable classifier

Although deep neural network approaches provide impressive results in sequence classification [9, 21, 69, 72], deep architectures are at least difficult to interpret. Therefore, many attempts are made to explain deep architectures [59]. However, it is claimed that restricting models to be interpretable does not necessarily lead to weaker performance and, hence, should be favored if possible [58, 82]. Moreover, particularly in the medical domain, knowledge regarding decision processes is strongly required for correct interpretation of the results [76].

Therefore, we focus on applying prototype-based methods using alignment-free dissimilarity measures for sequence comparison. In fact, prototype-based machine learning models for data classification and representation are known to be interpretable and robust [6, 82, 93]. Using such methods for the SARS-CoV-2 sequence data, first we verify the classification results for the GISAID data. In particular, we classify the sequences by a learning vector quantizer, which is proven to be robust and interpretable [60, 82]. Thereafter, we use this model to classify the new data from the NCBI. Moreover, this interpretable classifier provides correlation information regarding data features contributing to a class discrimination. This additional knowledge allows a further characterization of the virus classes. Additionally, the model is equipped with a reject option following [22]. This allows to refuse outliers by the model, which could give hints for new virus types.

## Materials and methods

### SARS-CoV-2 sequence databases in use

In order to investigate SARS-CoV-2 viruses in terms of sub-type spreading, two virus sequence datasets were considered.

#### The GISAID dataset $D_{G}$

The first one, abbreviated by $D_{G}$, is from the GISAID coronavirus repository (GISAID—Global Initiative on Sharing Avian Influenza Data). It consists by March 4, 2020, of 254 coronavirus genomes, isolated from 244 humans, nine Chinese pangolins, and one bat *Rhinolophus affinis*. After preprocessing, 160 complete human sequences are obtained as described in [23], where these genomes of SARS-CoV-2 have been used to create a phylogenetic network. The resulting network analysis distinguished three types of the virus (cluster) $A, B,\text { and } C: A$is most similar to the bat virus, whereas *B* and *C* are sequences obtained from *A* by two mutations: the synonymous mutation T8782C and the non-synonymous mutation C28144T changing a leucine to a serine. A further non-synonymous mutation G26144T changing a glycine to a valine lead from *B* to type *C*. In this sense, the classes (virus types) code implicitly the evolution in time of the virus.

In our data analysis, we removed two sequences, whose accession numbers occur twice in the data record, and another two, which we identified as not human resulting in 156 final sequences. Additionally, we take the type/class information as label for the virus genome sequences and, hence, as reference. A detailed data description as well as complete list of sequences can be found in [23]. The virus type assignments and additional data (country, collection date) as well as accession numbers for all 156 sequences in use are additionally provided in supplementary material.

The complete data information is found in supplementary files S12 Data.

#### The NCBI dataset $D_{N}$

The second dataset including 892 complete genomes has been selected from the National Center for Biotechnology Information (NCBI) Viral Genome database [10] and GenBank [14] by April 19, 2020, as given in Table 1. These data are human-based sequences and provide additionally the country information from which the sequences originate, as well as their collection date. For each sequence, we have also derived a more general assignment to regions based on the country information, which includes the following values: USA, China, Europe, and Others. The accession number and the additional data used in the analysis are included in supplementary material. We refer to this dataset by $D_{N}$.

Remark, although the SARS-CoV-2 virus is an RNA virus, the sequences provided by databases are given using the DNA coding. In the following, we take over this convention and do not explicitly refer to that later.

**Table 1:** Distribution of the NCBI data $D_N$ regarding regions and month of collection date

	China	Europe	USA	Others
December 2019	16	0	0	0
January 2020	44	4	16	9
February 2020	2	6	44	7
March 2020	1	23	706	10
April 2020	0	0	4	0

Again, the complete data information is found in supplementary files S12 Data.

### Representation of RNA sequences for alignment-free data analysis

Several approaches were published to represent sequences adequately for alignment-free comparison. These methods range from chaos game representation to standard unary coding or matrix representations. An overview is given in [84, 94, 95]. Here, we focus only on two of the most promising approaches—*Natural Vectors* and *Bag-of-Words*.

#### Natural vectors

*Natural Vectors* (NV) for nucleotide sequence comparison are based on a statistical sequence description for the distribution of nucleotide positions within a sequence ${\mathbf {s}}=\left[ s_,\ldots ,s_n\right]$ based on the alphabet ${\mathscr {A}}=\left\{ A,C,G,T\right\}$ [17, 42]. Let $\mu _{L}^{0}=n_{L}/n$ be the relative number (frequency) of the nucleotide $L\in {\mathscr {A}}$ and $p_{L}\left( j\right) /n$, $j=1\ldots n_{L}$ is the *relative* position of the *k*th nucleotide *L* in the sequence. Let $E\left[ r\right]$ further be the expectation operator of a random quantity *r*. With this convention, we get $\mu _{L}^{0}=E\left[ L\right]$ for the frequency of the nucleotide *L*. Further, we denote by $\mu _{L}=\mu _{L}^{1}=E\left[ p_{L}\right]$ the mean relative position of the nucleotide *L* in the sequence. The *k*th centralized moment $\mu _{L}^{k}$ for $k\ge 2$ is given as $\mu _{L}^{k}=E\left[ \left( p_{L}-\mu _{L}^{1}\right) ^{k}\right]$. Then, the natural vector of order *K* for a sequence ${\mathbf {s}}$ is defined as

1 $$\begin{aligned} {\mathbf {x}}\left( K,{\mathbf {s}}\right) =\left( \mu _{A}^{0},\mu _{C}^{0},\mu _{G}^{0},\mu _{T}^{0},\mu _{A}^{1},\mu _{C}^{1},\mu _{G}^{1},\mu _{T}^{1},\ldots ,\mu _{A}^{K},\mu _{C}^{K},\mu _{G}^{K},\mu _{T}^{K}\right) \end{aligned}$$

whereby we again drop the dependencies on *K* and ${\mathbf {s}}$ for simplicity, if it is not misleading.

Natural vectors are usually compared in terms of the $l_{p}$-metric

2 $$\begin{aligned} d_{p}\left( {\mathbf {x}},{\mathbf {y}}\right) =\root p \of {\sum _{j=0}^{K}\sum _{L\in {\mathscr {A}}}\left( \mu _{L}^{j}\left( {\mathbf {x}}\right) -\mu _{L}^{j}\left( {\mathbf {y}}\right) \right) ^{p}} \end{aligned}$$

giving the Euclidean distance for $p=2$. The Kendall statistics, as a kind of correlation measure, was used in [43].

The NV description of sequences can also be applied to nucleotide sequences containing ambiguous characters (degenerate bases) collected in the extension set ${\mathscr {E}}$ [15, 92]. This yields an extended alphabet ${\mathscr {A}}^{'}={\mathscr {A}}\cup {\mathscr {E}}$. In that case, weights $0\le w_{L}\left( s_{i}\right) \le 1$ are introduced for each $L\in {\mathscr {A}}$ with

$$\begin{aligned} w_{L}\left( s_{i}\right) ={\left\{ \begin{array}{ll} 1 &{} \text {if }s_{i}\in {\mathscr {A}}\wedge s_{i}=L\\ 0 &{} \text {if }s_{i}\in {\mathscr {A}}\wedge s_{i}\ne L\\ p_{L,s_{i}} &{} \text {otherwise} \end{array}\right. } \end{aligned}$$

where $p_{L,s_{i}}$ is the probability that the detected ambiguous character $s_{i}\in {\mathscr {E}}$ should be the character *L*. These weights have to be taken into account during the expectation value calculations [92].

#### Bag-of-words

Another popular method to compare RNA/DNA sequences is the method *Bag-of-words* (BoW) based on 3-mers, where the set *S* of words contains all possible 64 triplets defined by the nucleotide alphabet ${\mathscr {A}}=\left\{ A,C,G,T\right\}$ [7, 8, 21, 84]. Thus, all sequences ${\mathbf {s}}$ are coded as (normalized) histogram vectors of dimensionality $n=64$, such that we have for each sequence the corresponding histogram vector ${\mathbf {h}}\left( {\mathbf {s}}\right) \in {\mathbb {R}}^{n}$ with the constraints $h_{k}\left( {\mathbf {s}}\right) \ge 0$ and $\sum _{k=1}^{n}h_{k}\left( {\mathbf {s}}\right) =1$. Mathematically speaking, these vectors are discrete representations of *probability densities*. If the latter constraint is dropped, we have discrete representations of *positive functions*. The assignments of the triplets to the vector components $h_{i}$ are provided in supplementary material. If it is not misleading, we drop the dependence on ${\mathbf {s}}$ and simply write ${\mathbf {h}}$ instead of ${\mathbf {h}}\left( {\mathbf {s}}\right)$. As for NV, nucleotide sequences with ambiguous characters can be handled using appropriate expectation values.

Obviously, comparison of those histogram vectors can be done using the usual Euclidean distance. However, motivated by the already mentioned density property, an alternative choice is to compare them by means of divergence measures [46]. In the investigations presented later, we applied the Kullback–Leibler divergence [36]

3 $$\begin{aligned} D_{KL}\left( {\mathbf {h}},{\mathbf {m}}\right) =\sum _{j=1}^{n}h_{j}\cdot \log \left( h_{j}\right) -\sum _{j=1}^{n}h_{j}\cdot \log \left( m_{j}\right) \end{aligned}$$

for sequence histograms ${\mathbf {h}}$ and ${\mathbf {m}}$. Note that the first term in (3) is the negative Shannon entropy $H\left( {\mathbf {h}}\right) =-\sum _{j=1}^{n}h_{j}\cdot \log \left( h_{j}\right)$, whereas $Cr\left( {\mathbf {h}},{\mathbf {m}}\right) =\sum _{j=1}^{n}h_{j}\cdot \log \left( m_{j}\right)$ is the Shannon *cross-entropy*. Yet, other divergences like Rényi divergences could be used [85]. We refer to [79] for a general overview regarding divergences in the context of machine learning.

The assignment of the nucleotide triplets to the histogram dimension is found in supplementary material S13 Histogram Coding of Nucleotide Triplets.

### Machine learning approach for virus sequence data analysis

#### Median neural gas for data compression

The *Median Neural Gas* algorithm (MNG) is a neural data quantization algorithm for data compression based on (dis-)similarities [3, 16]. It is a stable variant of the *k*-median centroid method improved by neighborhood cooperativeness enhanced learning, where *k* is the predefined number of representatives [39, 49]. In this context, median approaches only assume a dissimilarity matrix for the data and restrict the data centroids to be data points. Thus, after training, MNG provides *k* data points to serve as representatives of the data. Thereby, the data space is implicitly sampled according to the underlying data density in consequence of the so-called magnification property of neural gas quantizers [48, 78].

It should be emphasized that despite the weak assumption of a given similarity matrix, MNG always delivers exact data objects as representatives. Hence, any averaging for prototype generation like in standard vector quantizers is avoided here. This is essential, if averaged data objects are meaningless like for texts, music data, or RNA/DNA sequences, for example.

#### Affinity propagation for clustering with cluster cardinality control

*Affinity propagation* (AP) introduced by Frey and Dueck in [24] is an iterative cluster algorithm based on message passing where the current cluster nodes, in the AP setting denoted as prototypes or exemplars, interact by exchanging real-valued messages. Contrary to methods like *c*-means or neural maps, where the number *c* of prototypes has to be chosen beforehand, AP starts assuming that all *N* data points are potential exemplars and reduces the number of valid prototypes (cluster centroids) iteratively. More precisely, AP realizes an exemplar-dependent probability model where the given similarities $\varsigma \left( i,k\right)$ between data points ${\mathbf {x}}_{i}$ and ${\mathbf {x}}_{k}$ (potential exemplars) are identified as log-likelihoods of the probability that the data points assume each other as a prototype. For example, the similarities $\varsigma \left( i,k\right)$ simply could be negative dissimilarities like the negative Euclidean distance.

The cost function $C_{AP}\left( I\right)$ minimized by AP is given by

$$\begin{aligned} C_{AP}\left( I\right) =-\sum _{i}\zeta \left( {\mathbf {x}}_{i},{\mathbf {x}}_{I\left( i\right) }\right) -\sum _{j}\delta _{j}\left( I\right) \end{aligned}$$

where $I:N\rightarrow N$ is the mapping function determining the prototypes for each data point given by means of

4 $$\begin{aligned} I\left( i\right) =\arg \max _{j}\left\{ a\left( i,k\right) +r\left( i,k\right) \right\} . \end{aligned}$$

and

$$ \delta _{j} \left( I \right) = \left\{ {\begin{array}{*{20}l}    { - \infty } \hfill & {{\text{if }}\exists \;j,k\;I\left( j \right) \ne j{\text{, }}I\left( k \right) = j} \hfill  \\    0 \hfill & {{\text{otherwise}}} \hfill  \\   \end{array} } \right. $$

is a penalty function. The quantity $r\left( i,k\right)$ is denoted as responsibility reflecting the accumulated evidence that point *k* serves as prototype for data point *i*. The availabilities $a\left( i,k\right)$ describe the accumulated evidence how appropriate data point *k* is seen as a potential prototype for the points *i*.

During the optimization, both kinds of messages are iteratively exchanged between the data by means of the alternating calculations according to

$$\begin{aligned} r\left( i,k\right) =\varsigma \left( i,k\right) -\max _{j\ne k}\left\{ a\left( i,j\right) +\zeta \left( i,j\right) \right\} \end{aligned}$$

and

$$ \begin{array}{*{20}l}    {a\left( {i,k} \right) = \min \left\{ {0,r\left( {k,k} \right) + \sum\limits_{{j \ne i,k}} {\max } \left\{ {0,r\left( {j,k} \right)} \right\}} \right\}} \hfill  \\    {a\left( {k,k} \right) = \max _{{j \ne k}} \left\{ {\max \left\{ {0,r\left( {j,k} \right)} \right\}} \right\}} \hfill  \\   \end{array}  $$

until convergence. Finally, the prototypes are determined according to (4).

Hence, $a\left( i,k\right)$ and $r\left( i,k\right)$ can be taken as log-probability ratios [24]. The iterative alternating calculation of $a\left( i,k\right)$ and $r\left( i,k\right)$ is caused by the max-sum-algorithm applied for factor graphs [54], which can further be related to spectral clustering [45].

The number of resulting clusters is implicitly determined by the self-similarities $\varsigma \left( k,k\right)$ also denoted as preferences. The larger the self-similarities the finer is the granularity of clustering [24]. Common choices are the median or the minimum of the similarities between all inputs. Otherwise, the self-similarities can be seen as a control parameter for the granularity of the clustering. Variation of this parameter provides information regarding stable cluster solutions in dependence of plateau regions of the resulting minimum cost function value.

#### The generalized learning vector quantizer: an interpretable prototype-based classifier

*Learning Vector Quantization* (LVQ) is an adaptive prototype-based classifier introduced by T. Kohonen [34]. A cost-function-based variant is known as *generalized LVQ* [62]. This cost function approximates the classification error [32]. In particular, an LVQ classifier requires training data $T=\left\{ \left( {\mathbf {x}}_{j},c\left( {\mathbf {x}}_{j}\right) \right) \in X\times {\mathscr {C}},\;j=1\ldots N\right\}$ where $X\subseteq {\mathbb {R}}^{n}$ and ${\mathscr {C}}=\left\{ 1,\ldots ,C\right\}$ is the set of available class labels. Further, the model assumes a set of prototypes $W=\left\{ {\mathbf {w}}_{k}\in {\mathbb {R}}^{n},\,k=1\ldots M\right\}$ with class labels $c\left( {{\mathbf {w}}_{k}}\right)$ such that at least one prototype is assigned to each class. Hence, we have a partitioning of the prototype set $W=\cup _{j=1}^{C}W_{j}$ with $W_{j}=\left\{ {\mathbf {w}}_{k}\in W|c\left( {\mathbf {w}}_{k}\right) =j\right\}$. Further, a dissimilarity measure $d\left( {\mathbf {x}},{\mathbf {w}}\right)$ is supposed, which has to be differentiable with respect to the second argument. For a given LVQ configuration, a new data point ${\mathbf {x}}$ is assigned to a class by the mapping

5 $$\begin{aligned} {\mathbf {x}}\mapsto c\left( {\mathbf {w}}_{\omega \left( W\right) }\right) \end{aligned}$$

with

6 $$\begin{aligned} \omega \left( W\right) =\mathsf {argmin}_{{\mathbf {w}}_{k}\in W}d\left( {\mathbf {x}},{\mathbf {w}}_{k}\right) \end{aligned}$$

is known as the winner-takes-all rule (WTA) in prototype-based vector quantization. The prototype ${\mathbf {w}}_{\omega }$ is denoted as winner of the competition.

During the learning, the cost-based LVQ minimizes the expected classification error $E_{X}\left[ E\left( {\mathbf {x}}_{k},W\right) \right]$ where

7 $$\begin{aligned} E\left( {\mathbf {x}}_{k},W\right) =f\left( \mu \left( {\mathbf {x}}_{k}\right) \right) \end{aligned}$$

is the local classification error depending on the choice of the monotonically increasing function *f* and the classifier function

8 $$\begin{aligned} \mu \left( {\mathbf {x}}_{k}\right) =\frac{d^{+}\left( {\mathbf {x}}_{k}\right) -d^{-}\left( {\mathbf {x}}_{k}\right) }{d^{+}\left( {\mathbf {x}}_{k}\right) +d^{-}\left( {\mathbf {x}}_{k}\right) }\in \left[ -1,1\right] \end{aligned}$$

where $d^{\pm }\left( {\mathbf {x}}_{k}\right) =d^{\pm }\left( {\mathbf {x}}_{k},{\mathbf {w}}^{\pm }\right)$ and ${\mathbf {w}}^{+}={\mathbf {w}}_{\omega \left( W_{c\left( {\mathbf {x}}_{k}\right) }\right) }$ is the so-called best matching correct prototype and ${\mathbf {w}}^{-}={\mathbf {w}}_{\omega \left( W\setminus W_{c\left( {\mathbf {x}}_{k}\right) }\right) }$ is the corresponding best matching incorrect prototype. Frequently, the squashing function *f* is chosen as sigmoid: $f_{\sigma }\left( z\right) =\frac{1}{1+\exp \left( -\sigma z\right) }$. Learning takes place as stochastic gradient descent learning (SGDL) [27, 57] of $E_{X}\left[ E\left( {\mathbf {x}}_{k},W\right) \right]$ with respect to the prototype set *W* to obtain an optimum prototype configuration in the data space.

The dissimilarity $d\left( {\mathbf {x}},{\mathbf {w}}\right)$ can be chosen arbitrarily supposing differentiability with respect to ${\mathbf {w}}$ to ensure SGDL. Frequently, the squared Euclidean distance $d_{E}\left( {\mathbf {x}},{\mathbf {w}}\right) =\left( {\mathbf {x}}-{\mathbf {w}}\right) ^{2}$ is applied resulting in the *standard generalized LVQ* (GLVQ). If both ${\mathbf {x}}$ and ${\mathbf {w}}$ are assumed as discrete representations of density functions, divergences like the Kullback–Leibler divergence $D_{KL}\left( {\mathbf {x}},{\mathbf {w}}\right)$ from (3) come into play instead [50]. It should be emphasized here that the non-symmetry of general divergences is not affecting the algorithm if it is used consistently in the predefined manner. Taking the variant $D_{KL}\left( {\mathbf {x}},{\mathbf {w}}\right)$ leads to the computational advantage that the Shannon entropy $H\left( {\mathbf {x}}\right)$ of the data according to (3) is not required to be calculated because the derivative with respect to a prototype ${\mathbf {w}}$ vanishes and, hence, does not contribute to the learning. In consequence, only the derivative of the cross-entropy $Cr\left( {\mathbf {x}},{\mathbf {w}}\right)$ affects the learning as it is also known from classification learning by deep neural networks [25].

The resulting LVQ variant is denoted as *divergence-based GLVQ* (GDLVQ). We refer to [79] for further considerations and mathematical analysis.

Another popular choice is the squared Euclidean mapping distance

9 $$\begin{aligned} d_{\varvec{\Omega }}\left( {\mathbf {x}},{\mathbf {w}}\right)=\, & {} \left( \varvec{\Omega }\left( {\mathbf {x}}-{\mathbf {w}}\right) \right) ^{2}\\ \nonumber \\=\, & {} \left( \varvec{\Omega }\left( {\mathbf {x}}-{\mathbf {w}}\right) \right) ^{T}\varvec{\Omega }\left( {\mathbf {x}}-{\mathbf {w}}\right) \nonumber \\ \nonumber \\=\, & {} \left( {\mathbf {x}}-{\mathbf {w}}\right) ^{T}\varvec{\Omega }^{T}\varvec{\Omega }\left( {\mathbf {x}}-{\mathbf {w}}\right) \nonumber \end{aligned}$$

proposed in [66] with the mapping matrix $\varvec{\Omega }\in {\mathbb {R}}^{m\times n}$ and *m* being the projection dimension usually chosen $m\le n$ [12]. Here, the data are first mapped linearly by the mapping matrix and then the Euclidean distance is calculated in the mapping space ${\mathbb {R}}^{m}$. The mapping matrix can be optimized again by SGDL to achieve a good separation of the classes in the mapping space. The respective algorithm is known as *Generalized Matrix LVQ* (GMLVQ) [65]. Note that SGDL for $\varvec{\Omega }$-optimization usually requires a careful regularization technique [64].

After training, the adapted projection matrix $\varvec{\Omega }$ provides additional information. The resulting matrix $\varvec{\Lambda }=\varvec{\Omega }^{T}\varvec{\Omega }\in {\mathbb {R}}^{n\times n}$ allows an interpretation as *classification correlation matrix*, i.e., the matrix entries $\Lambda _{ij}$ give only those correlation information between data features *i* and *j*, which contribute to the class discrimination [5, 77]. Thus, it is not comparable with the data correlation matrix (or covariance), which does not reflect class discriminating correlations. Moreover, because the $\varvec{\Omega }$-matrix, and therefore also the $\varvec{\Lambda }$-matrix, is optimized to maximize the classifier accuracy, bias effects as known from covariance estimation as explained in [19] are not problematic in this context.

Instead of the linear $\varvec{\Omega }$ mapping, nonlinear mappings could be considered explicitly as suggested in [81] or implicitly by means of kernel distances [63, 80].

A trained LVQ model can be applied to newly incoming data of unknown distribution. However, care must be taken to ensure that the model remains applicable and that there is no inconsistency with the new data. Therefore, each LVQ can be equipped with a reject option for the application phase [22, 29]. If the dissimilarity of the best matching prototype to a data point is greater than a given threshold $\tau$, it is refused for classification, i.e., this optional tool equips the LVQ with a so-called *self-controlled evidence* (SCE) [82]. The threshold $\tau$ is determined during model training for each prototype individually, e.g., $95\%$ percentile of the dissimilarity value for those data, which are assigned to the considered prototype by the WTA rule (6) together with the class assignment (5).

In fact, this reject option improves the robustness of the model [61].

### Stochastic neighbor embedding for visualization

The method of stochastic neighbor embedding (SNE) was developed to visualize high-dimensional data in a typically two-dimensional visualization space [30]. For this purpose, each data point ${\mathbf {x}}_{k}$ in the data space is associated with a visualization vector ${\mathbf {v}}_{k}\in {\mathbb {R}}^{2}$. The objective of the respective embedding algorithm is to distribute the visualization data in a way that the density of original data distances in the high-dimensional data space is preserved as good as possible for the respective density of the distances in the visualization space (embedding space). The quality criterion is the Kullback–Leibler divergence between them, which is minimized by SGDL with respect to the visualization vectors ${\mathbf {v}}_{k}$.

Yet, SNE suffers from the fact that the distance densities in the original data space are frequently heavy-tailed [11], which leads to inaccurate visualizations. To overcome this problem, the so-called *t*-*distributed* SNE (*t*-SNE) was developed [74].

### Data processing workflow

In the following, we describe and motivate the steps of data processing and analysis.

1. Coding of all sequences of $D_{G}$ data and $D_{N}$ data.
  Alphabet ${\mathscr {A}}^{'}={\mathscr {A}}\cup {\mathscr {E}}$ with alphabet extension ${\mathscr {E}}=\left\{ B,D,H,K,M,N,R,S,V,W,Y\right\}$ due to ambiguous characters in the datasets.
  A natural vector representation ${\mathbf {x}}\left( 4,{\mathbf {s}}\right) \in {\mathbb {R}}^{20}$ of order $K=4$ is generated for each sequence ${\mathbf {s}}$ according to (1) paying attention to the alphabet extension ${\mathscr {E}}$.
  A BoW-representation for 3-mers is generated for each sequence ${\mathbf {s}}$: ${\mathbf {h}}\left( {\mathbf {s}}\right) \in {\mathbb {R}}^{64}$ according to the possible nucleotide triplets of the alphabet ${\mathscr {A}}=\left\{ A,C,G,T\right\}$ paying attention to the alphabet extension ${\mathscr {E}}$
2. Training of LVQ-classifiers for $D_{G}$ data to evaluate the results from [23] obtained by phylogenetic trees
  Training data are all samples of $D_{G}$ with the additional virus type assignment *A*, *B*, or *C* taken as class labels.
  For all LVQ variants, we take only one prototype per class.
  For GMLVQ, the projection matrix is chosen as $\varvec{\Omega }\in {\mathbb {R}}^{2\times n}$, i.e., the mapping dimension is $m=2$.
  SGDL training as tenfold cross-validation to determine the best LVQ architecture for the given problem.
    Training of *W* using the GLVQ for NV representation.
    Training of *W* and $\varvec{\Omega }$ using the GMLVQ for NV representation.
      GDLVQ is not applicable for this sequence representation due to mathematical reasons.
    Training of *W* using the GLVQ for BoW representation.
    Training of *W* and $\varvec{\Omega }$ using the GMLVQ for BoW representation.
  Final training of the best LVQ architecture with optimum training schedule to achieve best prototype configuration *W*.
    If GMLVQ architecture is selected for final training: training of both *W* and $\varvec{\Omega }$, determination of the classification correlation matrix $\varvec{\Lambda }=\varvec{\Omega }^{T}\varvec{\Omega }$.
    Determination of the reject thresholds for each prototype for self-controlled evidence use based on the $95\%$ percentile rule.
3. Clustering $$D_{N}$$ data
  Compression of the subset of 706 US sequences of March by MNG to achieve 50 representatives by MNG using 50 prototypes.
  Generating a balanced subset consisting of all China samples (63), all Europe samples (33), and USA samples (114) for cluster analysis. The US samples comprise the 50 representatives from MNG and all US samples from January and February. The samples from other regions are not considered for cluster analysis. We denote this balanced dataset extracted from $D_N$ by $D_{NB}$.
  Clustering and identification of stable cluster solutions using affinity propagation by means of the control parameter $\varsigma =\varsigma \left( k,k\right)$ $\forall k$.
4. Classification of the $D_{NB}$ data as well as the full $D_N$ data using the best LVQ classifier with integrated self-controlled evidence
  Classification of the $D_{NB}$ data by the final LVQ classifier with reject option using the determined thresholds to realize the self-controlled evidence (SCE).
  Evaluation of the data rejected by the SCE rule.

## Results

According to the processing workflow, we trained several LVQ classifier variants for the $D_{G}$ data. By tenfold cross-validation, we achieved the averaged accuracies depicted in Table 2 together with their respective standard deviations. According to these results, GMLVQ performs best using the BoW coding of the sequences together with the Euclidean mapping distance $d_{\varvec{\Omega }}\left( {\mathbf {x}},{\mathbf {w}}\right)$ from (9). Thus, we finally trained a GMLVQ network for both the prototype set *W* containing one prototype per class and the mapping matrix $\varvec{\Omega }$ using the sequence BoW coding. For this final network, a classification accuracy of $100\%$ is obtained while rejecting seven samples for classification according to the SCE decision. The resulting classification correlation matrix $\varvec{\Lambda }=\varvec{\Omega }^{T}\varvec{\Omega }$ is depicted in S1 Fig. Because $\varvec{\Omega \in {\mathbb {R}}^{2\times n}}$, it can serve for a data mapping into a two-dimensional visualization space. Accordingly, all $D_{G}$ data together with the GMLVQ prototypes are visualized in S2 Fig. An additional visualization of the learned prototypes is given in S3 Fig.

The list of rejected sequences is provided in supplementary material $\hbox {S14 GMLVQ Mapping for D}_N$.

The clustering of the $D_{NB}$ dataset suggests cluster solutions with either 2, 4, or 5 clusters according to the stability range of the control parameter $\varsigma$, as shown in S4 Fig. We visualized the four-cluster solution using the *t*-SNE as depicted in S5 Fig. The respective cluster centroids are visualized in S6 Fig.

Applying the trained GMLVQ classifier to the $D_{NB}$ dataset leads to the classification of 37 data points to class *A*, 95 data points to class *B*, and 2 data points to class *C*. According to the SCE decision, 59 data points were rejected from classification by the learned GMLVQ classifier. The result is given in S7 Fig using the *t*-SNE as visualization scheme. The visualization of the classification result by means of the $\varvec{\Omega }$ mapping from the GMLVQ model delivers S8 Fig.

The distribution of the sequence data from the $D_{NB}$ dataset with respect to the geographic sequence origins (regions) and the respective collection dates together with the class assignments is presented in S9 Fig. A respective visualization of the distribution for the dataset $D_G$ is shown in S10 Fig.

The classification of the full $D_N$ dataset assigns 154 data points to class *A*, 293 data points to class *B*, and 20 data points to class *C*, whereas 495 data points are rejected according to the SCE rule. The class assignments are visualized in S11 Fig.

**Table 2:** Classification results of trained LVQ variants for the $D_{G}$ dataset obtained by tenfold cross-validation

	NV		BoW		
	GLVQ	GMLVQ	GLVQ	GDLVQ	GMLVQ
Averaged accuracy	$53.1\%$	$56.4\%$	$81.7\%$	$87.7\%$	$97.4\%$
Standard deviation	$\pm 9.8\%$	$\pm 6.3\%$	$\pm 4.4\%$	$\pm 6.2\%$	$\pm 1.5\%$

The predicted virus type or the rejection decision for each sequence from $D_N$ according to the GMLVQ class assignment or the SCE decision is found in supplementary material $\hbox {S14 GMLVQ Mapping for D}_N$.

## Discussion

The classification analysis of the $D_{G}$ data by means of the machine learning model GMLVQ verifies the class determination suggested in [23]. Only seven data samples are not classified accordingly due to the model self-controlled evidence decision. Thereby, the GMLVQ model shows a stable performance in learning (Table 2), which underlines its well-known robustness [60]. Thus, we observe an overall precise agreement supporting the findings in [23].

This agreement, however, is obtained by alignment-free sequence comparisons. More precisely, the nucleotide-based BoW sequence coding delivers a perfect separation of the given classes for the learned mapping distance $d_{\varvec{\Omega }}\left( {\mathbf {x}},{\mathbf {w}}\right)$.

Yet, the computational complexity of a single dissimilarity calculation for the encoded sequences is only $O\left( 64 \cdot m \cdot N_W \right)$ with $m=2$ being the mapping dimension of $\varvec{\Omega }$ and $N_W=|W|$ is the number of all prototypes in GLVQ/GMLVQ. The overall BoW sequence coding takes $O\left( l \cdot N\right)$. Paying attention to the fact that the GLVQ/GMLVQ training time scales with the number *N* of data, we have an overall complexity of $O\left( 64\cdot m \cdot N_W \cdot N\right)$ for model learning based on the coded data. Together with the time complexity $O\left( l \cdot N \right)$ for BoW-coding of all data with the sequence length *l*, we finally obtain an overall complexity of $O\left( N \cdot (64\cdot m \cdot N_W +l) \right)$ which usually is much lower than $O(N^4 + N \cdot l^2)$ for alignment-based methods [18], because $N_W\ll N$ and $n\ll l$ is valid.

Further, because GMLVQ is an interpretable classifier, we can draw further conclusions from the trained model: The resulted classification correlation matrix $\varvec{\Lambda }$ depicted in S1 Fig suggests that particularly the histogram dimensions 27 and 28 are important in correlation with the other dimensions. These dimensions refer to the frequency of the triplets “CGG” and “CGT” in the sequences. Moreover, both dimensions should be negatively correlated for good class separation. This discrimination is a key feature of GMLVQ. Although the prototypes look very similar, as shown in S3 Fig, the $\varvec{\Omega }$ is sensitive to smallest deviations in the histograms. Yet, we cannot expect greater deviations, because the sequences differ only in few characters according to the special mutations [23, 28]. The AP centroids differ slightly more than the GMLVQ prototypes, as shown in S6 Fig. This can be dedicated to larger overall scattering of the $D_{NB}$ data.

Further, the GMLVQ prototypes serve as class “detectors.” If the encoded sequences are most similar to them with respect to the mapping distance, the sequences are assigned to the respective classes according to the WTA rule (6). However, in general the prototypes are not identical with the mean vectors of the class distribution, as emphasized in [52].

Application of the GMLVQ to the $D_N$ and $D_{NB}$ data from the NCBI offers new insights. First, coloring of the data in the *t*-SNE visualization S7 Fig of $D_{NB}$ according to the obtained class assignments seems to be confusing: The classes cannot be detected as separate regions in that case. However, applying the $\varvec{\Omega }$ mapping S8 Fig, the class structure becomes visible also for this dataset. The reason for this discrepancy could be that both *t*-SNE and AP implicitly reflect data densities in the data space. Class densities, however, do not have to coincide with the overall data density. Thus, the $\varvec{\Omega }$ mapping, which is optimized during GMLVQ training for best classification performance, offers the better visualization option and, hence, disclosures the class distribution more appropriately.

Comparing the class distributions of the sequences with respect to origins (regions) and collection dates for $D_{NB}$ in S9 Fig and $D_G$ in S10 Fig, both class distributions within the cells show a similar behavior. The $D_{NB}$ dataset from NCBI contains only a few samples from Europe, all occurring from February onward, i.e., no European data samples from December/January were available. We observe that class *C* for the $D_G$ data is mainly represented in January for European samples, which confirms the findings in [23]. Thus, the small number of class *C* samples in the $D_{NB}$ classification may be addressed to this peculiarity in Europe. Further, the GMLVQ, which was trained by $D_G$ data, rejects a large amount of data from $D_{NB}$, particularly in March. We suspect an accumulation of mutations which could explain the scattering. Accordingly, the GMLVQ is able to detect this behavior by means of the SCE decision rule.

We observe from the visualization S11 Fig of the classification for the $D_N$ data that the data points rejected for classification scatter around the dense class regions. Thus, we can conclude that the nucleotide base mutations in the viral sequences, which cause the scattering, do not show a new coherent profile, at least at this time.

## Conclusion

In this contribution, we investigate the application of interpretable machine learning methods to identify types of SARS-CoV-2 virus sequences based on alignment-free methods for RNA sequence comparison. In particular, we trained a *generalized matrix learning vector quantizer* classifier model (GMLVQ) for a dataset with given virus type information, which was obtained by phylogenetic tree analysis [23]. GMLVQ supposes vectorial data representations and compares vectors in terms of a well-defined dissimilarity measure. In this application, the GMLVQ training is based on the Bag-of-Words coded sequences and yields class specific prototype vectors as well as an optimum class/typus separating dissimilarity measure in the data space of encoded sequences. Compared to phylogenetic trees or multiple sequence alignment, which require high computational costs due to the involved sequence alignment process, the GMLVQ approach has lower complexity and allows an easy out-of-training generalization.

By means of the trained GMLVQ, we first verified the SARS-CoV-2 virus types determined in this first dataset. Further, considering a classification correlation matrix delivered by GMLVQ optimization, we are able to identify features which contribute decisively to a type separation.

Second, we applied the trained GMLVQ to another dataset obtained from the NCBI database without virus type information. Using the self-controlled evidence property of the GMLVQ, we are able to classify these sequences to the previously identified types, avoiding the application of the model to inconsistent data compared to the training data. Further, the rejected data allow speculations about new virus types with respect to nucleotide base mutations in the viral sequences.

Yet, an appropriate training and data coding for successful GMLVQ application require a careful and precise data handling as well as model training regime, i.e., respective expert knowledge.

Future work will consider the replacement of the WTA rule (6) by a fuzzy variant (winner ranking) resulting in a probabilistic class/type assignment instead of the crisp rule (5). This probabilistic view could be further integrated into the SCE-based rejection decision to differentiate between rejected sequences regarding their consistence to the GMLVQ version in use. Thus, the user can decide whether to retrain the model adding a new class or continue with the current configuration.

## Supplementary Information

Below is the link to the electronic supplementary material.

Supplementary material 1 (pdf 601 KB)

Supplementary material 2 (XLSX 45KB)

Supplementary material 3 (XLSX 10 KB)

Supplementary material 4 (XLSX 25KB)
