# Supplementary material for: Learning vector quantization as an interpretable classifier for the detection of SARS-CoV-2 types based on their RNA sequences
Source: Neural Comput Appl. 2021 Apr 27;34(1):67–78. doi: 10.1007/s00521-021-06018-2 (PMC8076884; doi:10.1007/s00521-021-06018-2)
Supplement: Supplementary file 1 — Supplementary material 1 (pdf 601 KB) [file 521_2021_6018_MOESM1_ESM.pdf]

Figures

S1 Fig.

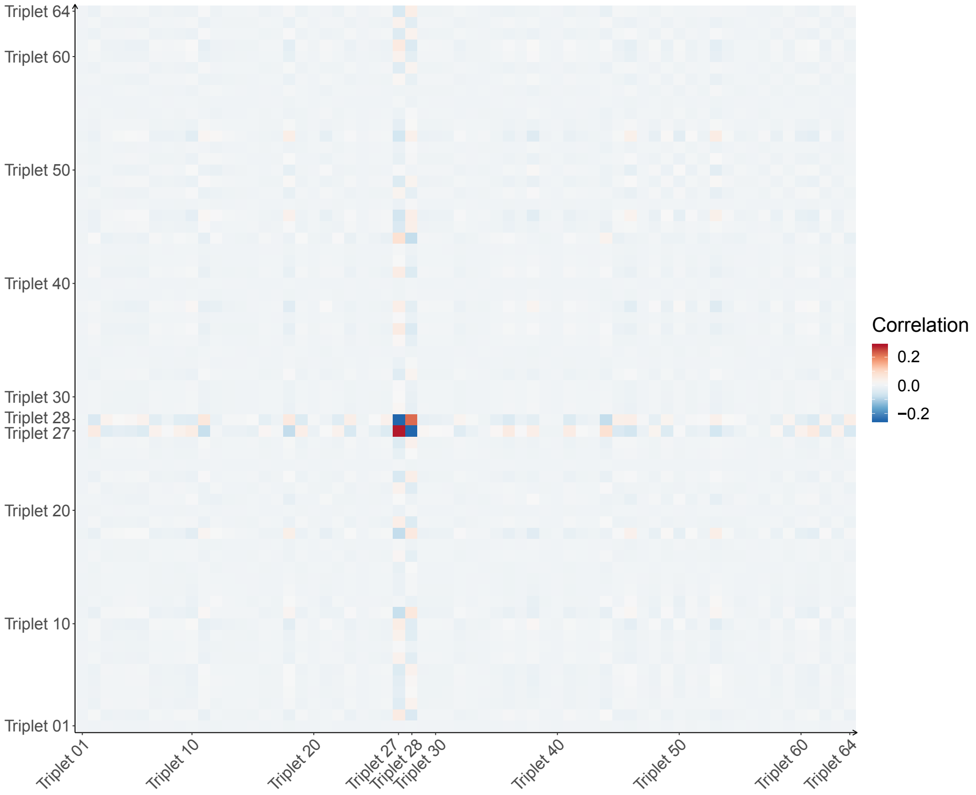

Visualization of the classification-correlation matrix. The classification-correlation matrix provides the linear correlations among the triplet frequencies of the sequences, which contribute to the class discrimination.

S2 Fig.

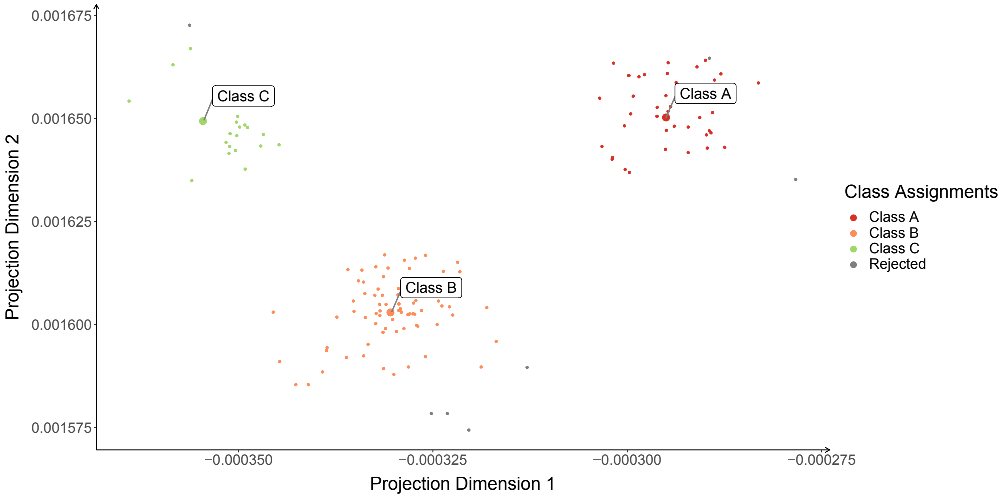

Visualization of the GMLVQ result for D_G-data. The data as well as the the GMLVQ-prototypes are mapped using the learned $\mathbf{\Omega}$-matrix. The data points are colored either regarding their class assignments or regarding their reject decision. The GMLVQ-prototypes serve as class representatives. However, they are not identical with the mean vectors of the classes.

S3 Fig.

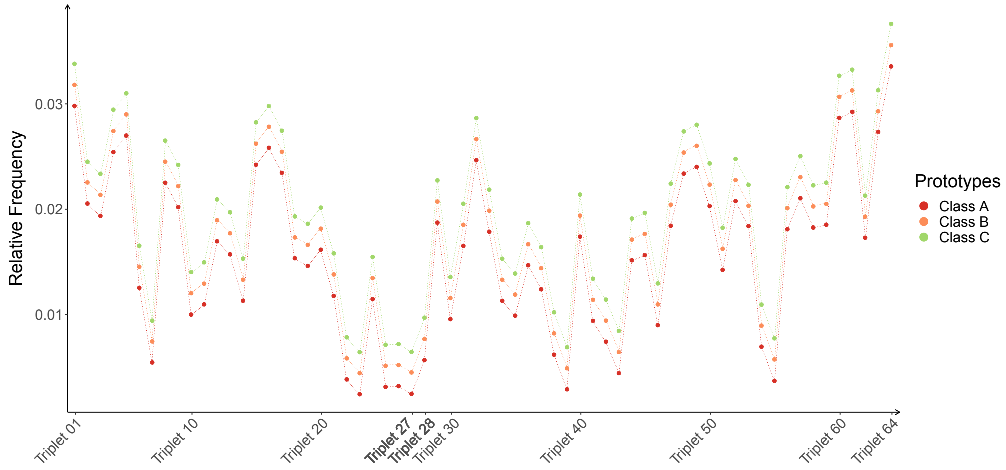

Visualization of the GMLVQ prototypes. The color of the prototypes is in agreement with the class coloring in S2 Fig. Further, the prototypes are vertically shifted by small offsets for better visualization and separation.

S4 Fig.

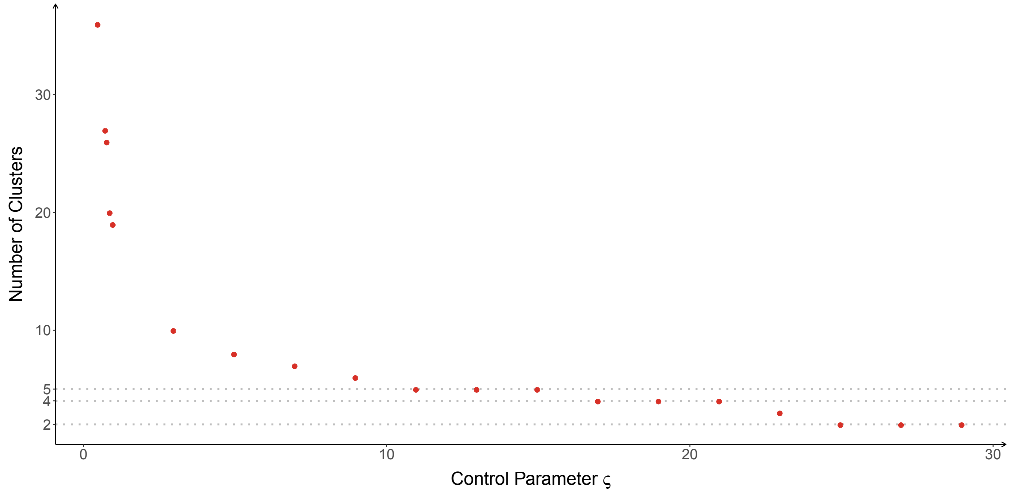

Stability of AP cluster solutions for D_{NB}-data. The number of clusters in dependence on the control parameter ς is depicted. Plateaus refer to stable cluster solutions. Accordingly, we identify 2-, 4-, and 5-cluster solutions as most recommended.

S5 Fig.

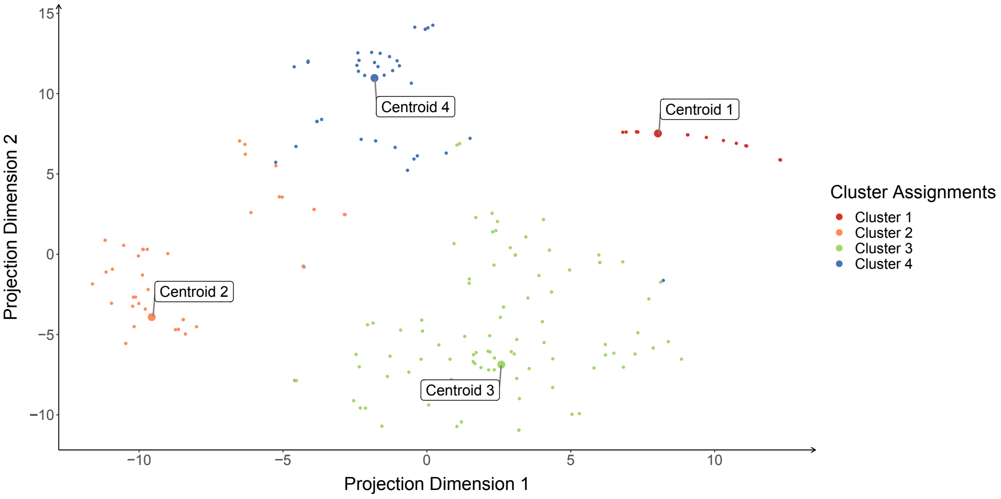

Visualization of the AP clustering result for D_{NB}-data using 4 clusters. The data as well as the cluster centroids are depicted using the t-SNE.

S6 Fig.

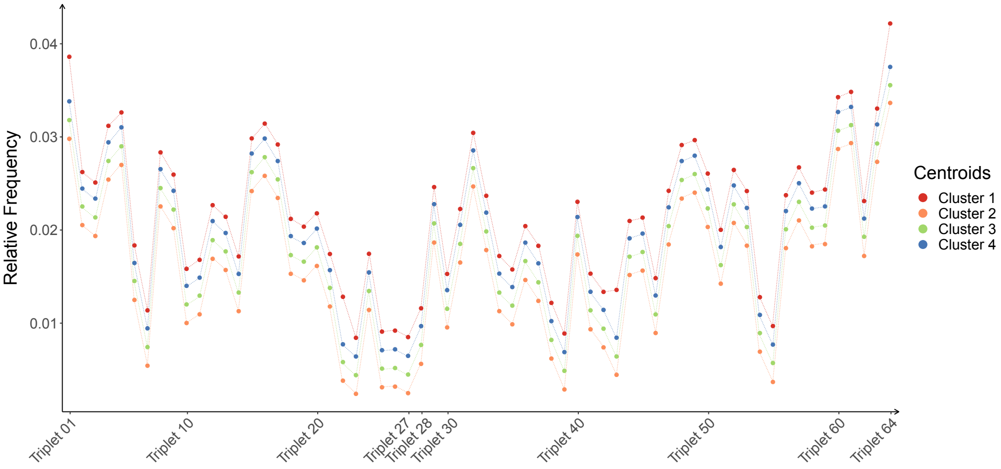

Visualization of the AP cluster centroids for D_{NB}-data using the 4-cluster solution. The color of the cluster centroids is in agreement with the cluster coloring in S5 Fig. Further, the centroids are vertically shifted by small offsets for better visualization and separation.

S7 Fig.

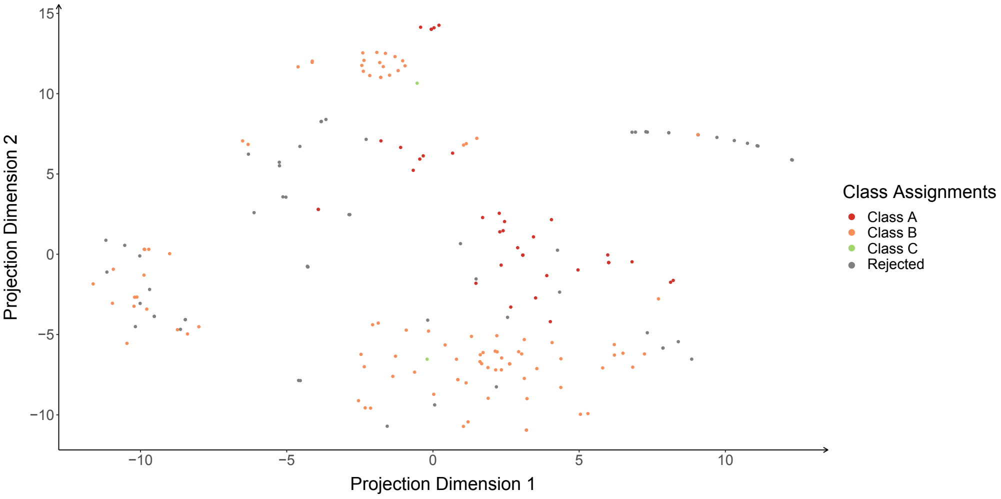

Visualization of GMLVQ classification for the D_{NB}-data by t-SNE. The class coloring is as in S2 Fig.

S8 Fig.

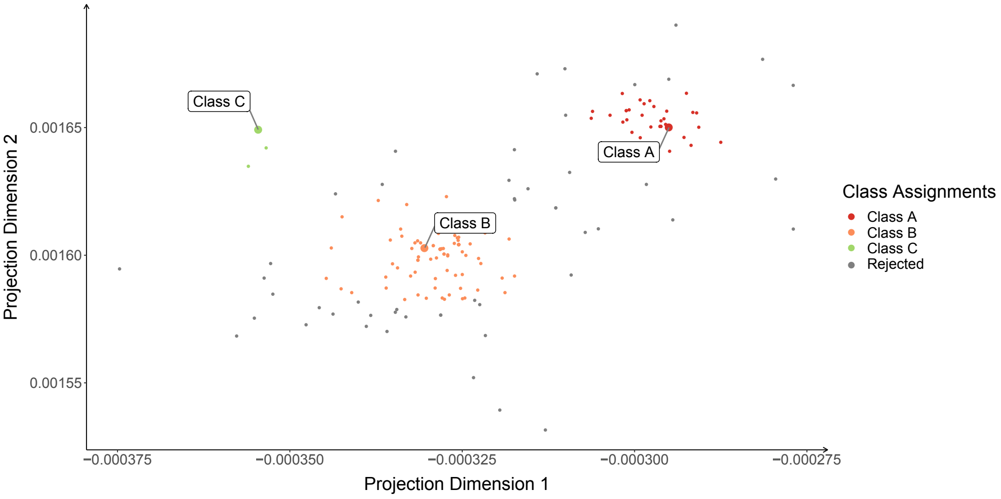

Visualization of GMLVQ classification for the D_{NB}-data by Ω-mapping. The data as well as the GMLVQ prototypes are depicted. The class coloring is as in S2 Fig.

S9 Fig.

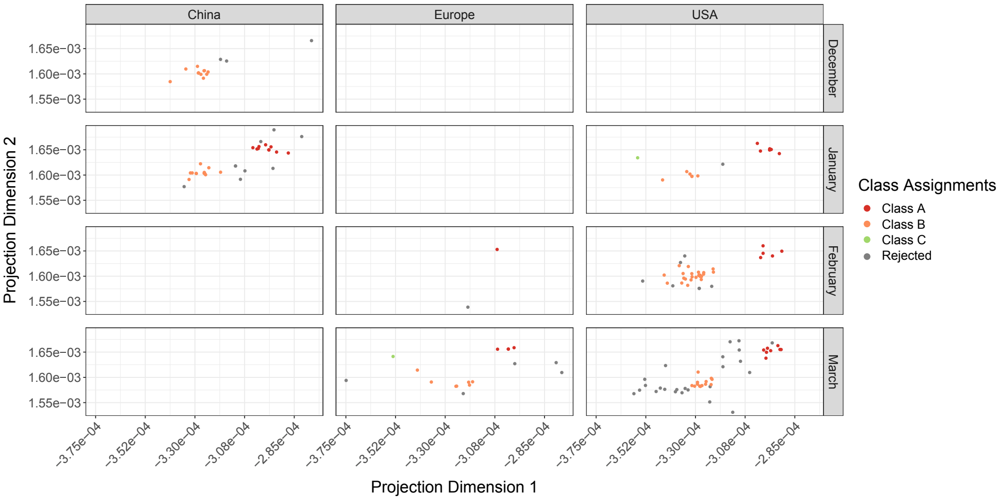

Distribution of the D_{NB}-data depending on the geographic origin and the collection date. A distribution of the data from the D_{NB}-dataset with respect to the geographic sequence origin and the collection date together with the class assignments. Again, the mappings are realized by the $\mathbf{\Omega}$ matrix. The class coloring is as in S2 Fig.

S10 Fig.

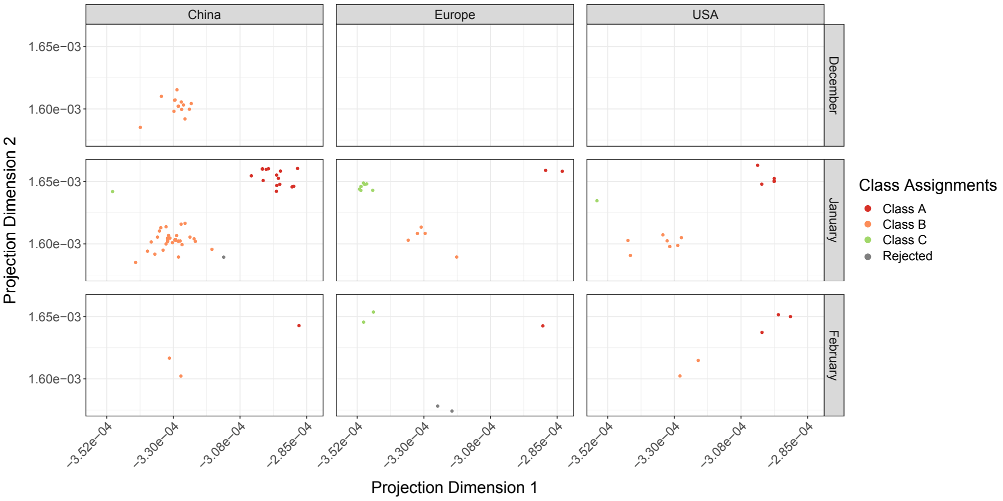

Distribution of the D_G-data depending on the geographic origin and the collection date. A distribution of the data from the D_G-dataset with respect to the geographic sequence origin and the collection date together with the class assignments. Again, the mappings are realized by the $\mathbf{\Omega}$ matrix. The class coloring is as in S2 Fig.

S11 Fig.

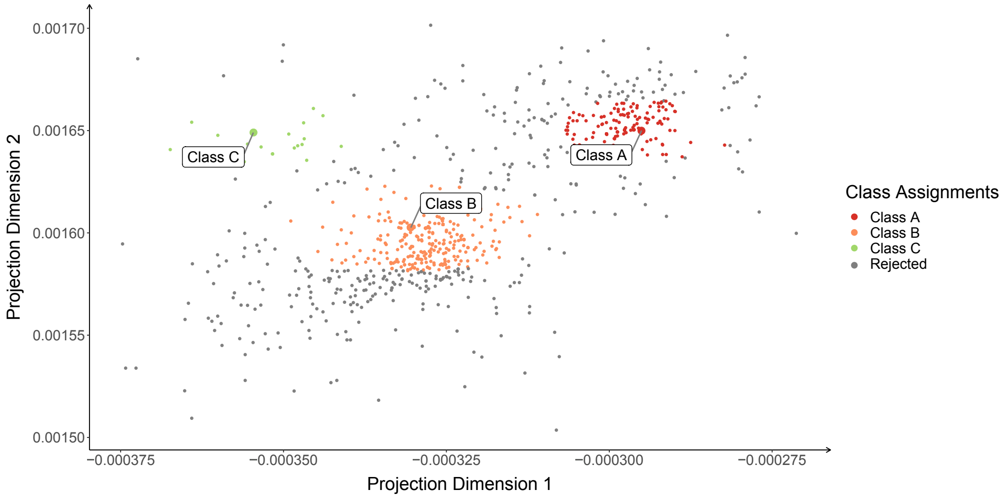

Visualization of GMLVQ classification for the full D_N-data by Ω-mapping. The data as well as the GMLVQ prototypes are depicted. The class coloring is as in S2 Fig.

S12 Data Data Files The data file 'S12 Data.xlsx' (Excel) contains the accession numbers of both datasets D_G and D_N. Further collection date and origin (region) are attached. For the D_G dataset, additionally, the type information (class assignment) is given.

S13 Histogram Coding of Nucleotide Triplets Assignment of the histogram dimensions to the nucleotide triplets For each of the 64 nucleotide combinations, the coding by the histogram dimensions is given in the file 'S13 Histogram Coding.xlsx'.

S14 GMLVQ Mapping for D_N Virus type assignments for the D_N-sequences obtained by GMLVQ For each sequence in D_N, the class/type assignment obtained by the GMLVQ model is given as well as if a rejection decision was made according to the SCE of the GMLVQ model. Additionally, we provide the sequences from D_G, which were rejected by the SCE decision of the GMLVQ model. The respective file is 'S14 GMLVQMapping.xlsx'.
